# Urgent development of effective therapeutic and prophylactic agents to control the emerging threat of Middle East respiratory syndrome (MERS)

**DOI:** 10.1038/emi.2015.37

**Published:** 2015-06-17

**Authors:** Lu Lu, Shuai Xia, Tianlei Ying, Shibo Jiang

**Affiliations:** Key Laboratory of Medical Molecular Virology of Ministries of Education and Health, Shanghai Medical College and Institute of Medical Microbiology, Fudan University, Shanghai 200032, China

**Dear Editor,**

As of June 12, 2015, the World Health Organization had been notified of 1289 laboratory-confirmed cases of Middle East respiratory syndrome coronavirus (MERS-CoV) infection globally, including at least 455 related deaths (case-fatality rate of 35%) (http://www.who.int/csr/don/12-june-2015-mers-korea/en/). On May 20, a 68-year-old man returning to the Republic of Korea from a trip to Saudi Arabia was diagnosed with MERS-CoV infection, and since then, the virus has spread, resulting in a cluster of 145 MERS-CoV cases, including 14 deaths, in South Korea and China (http://www.who.int/csr/disease/coronavirus_infections/en/), bringing the total number of countries reporting MERS-CoV infection to 25. This patient became the one of the most active “MERS super-spreader”, while the MERS super-spreader in Abu Dhabi was linked to 28 confirmed MERS cases (http://www.recombinomics.com/News/04291401/UAE_MERS_Superspreader_28.html).

Most of these cases in this cluster represent medical staff, family and other caregivers, or those close to the original patient before he was diagnosed with MERS-CoV infection and isolated. Therefore, an understandable sense of panic has arisen among those who have had contact with the newly diagnosed cases. This is yet another alarm sounding the necessity for the rapid development of therapeutic and prophylactic agents to treat MERS patients and protect high-risk populations from MERS-CoV until an effective and safe vaccine is available.^1,2^

Based on our previous experience in developing viral fusion inhibitors against HIV^3^ and SARS-CoV,^4^ we designed and synthesized a peptide (HR2P) derived from the HR2 domain in the S2 subunit of the spike (S) protein of the MERS-CoV EMC/2012 strain. We found that HR2P could bind with the HR1 domain to form a stable six-helix bundle and thus inhibit viral fusion core formation and S protein-mediated cell-cell fusion. HR2P was demonstrated to potently inhibit infection by both pseudotyped and live MERS-CoV in different cell lines.^5^ We then modified the HR2P peptide by introducing Glu (E) and Lys (K) residues at the *i* to *i*+4 or *i* to *i*+3 arrangements. We found that one of these HR2P analogous peptides, HR2P-M2, exhibited significantly improved stability, solubility and antiviral activity.^5^

Interestingly, the HR2P-M2 peptide could potently inhibit infection by pseudoviruses expressing MERS-CoV S protein with or without mutation in the HR1 region, suggesting that it could be effective against most currently available MERS-CoV mutants. We further demonstrated that the HR2P-M2 peptide administered via the intranasal route could protect Ad5-hDPP4-transduced mice^6^ from challenge by MERS-CoV strains with or without mutations in the HR1 region, indicating that this peptide could be used as a nasal spray to protect high-risk populations, including healthcare workers, MERS patients' family members, and those having close contacts with the patients, from MERS-CoV infection.^7^ Intranasal application of the peptide to MERS-CoV-infected patients may suppress viral replication in epithelial cells of the respiratory tract and thus reduce the release of virions, thereby preventing the spreading of MERS-CoV to other people.

Furthermore, we and other groups have identified monoclonal antibodies (mAbs) targeting neutralizing epitopes in the receptor-binding domain of the S1 subunit of MERS-CoV S protein. These mAbs, including m336,^8^ MERS-4,^9^ 3B11,^10^ and Mersmab1,^11^ have shown potent neutralizing activity against MERS-CoV. The human mAb m336, which was derived from a very large naive antibody library, belongs to the IgG1 subclass, with high avidity (99 pM) and neutralizing activity against pseudotyped and authentic MERS-CoV (the half maximal inhibitory concentration of 0.033 and 0.47 nM, respectively).^12^ More interestingly, the m336 mAb is a germline-like antibody with only one mutation in the heavy chain and five in the light chain. As such, it is safe for humans, highly expressible, and highly soluble. *In vivo* studies have shown that this mAb is very effective in protecting MERS-CoV-susceptible animals from viral challenge (unpublished data), suggesting that the m336m mAb is a very promising drug candidate for the urgent treatment of MERS-CoV-infected patients.^12^

We have also performed *in vitro* studies demonstrating that the combination of HR2P-M2 peptide with m336 mAb exhibited a strong synergistic effect against MERS-CoV infection (unpublished data). This observation suggests that intranasal administration of HR2P-M2 peptide combined with intravenous administration of m336 mAb may be a powerful strategy for treatment of MERS patients.

Laboratory-produced mAbs m102.4, a human mAb against Hendra virus and Nipah virus, and Zmapp, comprising three chimeric mAbs against Ebola virus, have shown good *in vivo* efficacy in animal models^13,14^ and have been successfully used in clinics to treat patients infected by Hendra virus or Nipah virus^13^ and Ebola virus,^15^ respectively. Therefore, it can be plausibly suggested that m336 mAb and HR2P-M2 peptide, both of which have demonstrated excellent *in vivo* efficacy in animal models, may also have high potential for clinical application in both urgent and prophylactic treatment of MERS patients.
